# A review of the synergistic mechanisms of Yijinjing, meridian sinew therapy, dry needling, and Fascial Manipulation in chronic cervical myofascial pain based on the fascia–meridian sinew continuum

**DOI:** 10.3389/fmed.2026.1881739

**Published:** 2026-07-23

**Authors:** Mengyao Liu, Yongkai Li, Rong Bian

**Affiliations:** 1 Hospital of Traditional Chinese Medicine Affiliated to Xinjiang Medical University, Urumqi, China; 2 School of Health Management, Xinjiang Medical University, Urumqi, China

**Keywords:** chronic cervical myofascial pain, dry needling, fascial continuum, Fascial Manipulation, integrative rehabilitation, meridian sinew theory, narrative review, non-pharmacological intervention

## Abstract

Chronic cervical myofascial pain is a common and disabling musculoskeletal pain condition, yet its persistence is unlikely to be explained by a single local lesion. Current evidence points to a multilevel process involving hypersensitive nodal sites, impaired fascial gliding, imbalance across continuous tension chains, sensorimotor disturbance, and mechanisms that maintain pain chronicity. Alongside advances in fascial science and mechanobiology, traditional meridian sinew theory offers a clinically useful language for pathway-based pain, tension-chain imbalance, remote–local interaction, and functional restriction. This narrative review with expert synthesis examines possible correspondences between meridian sinew theory and the modern fascial continuum and considers the layer-specific roles of Yijinjing, meridian sinew therapy, dry needling, and Fascial Manipulation in chronic cervical myofascial pain. These correspondences are framed as clinical heuristics and phenomenological analogies, not as anatomical equivalences or proven mechanistic homologies. We propose a hypothesis-driven staged framework in which dry needling may assist local desensitization, Fascial Manipulation and meridian sinew therapy may support chain correction, and Yijinjing may contribute to active remodeling and long-term self-management. The framework is intended to guide research and clinical reasoning, not to serve as a validated treatment algorithm. We also discuss transferability to primary care and non-TCM systems, safety and contraindications, evidence certainty, intervention standardization, objective mechanistic measures, and priorities for future studies, including staged trials, health-economic evaluation, patient-reported experience measures, and wearable sensor-based monitoring.

## 1 Introduction

Neck pain is one of the most common musculoskeletal disorders worldwide and a major cause of long-term functional limitation and disability. Analyses from the Global Burden of Disease Study 2021 estimated that the number of cases of neck pain would reach approximately 269 million by 2050, representing an increase of 32.5% from 2020 and highlighting the need for durable management strategies rather than short-term symptom control (1). In clinical practice, chronic cervical myofascial pain usually presents with recurrent pain, local stiffness or tightness, restricted movement, and referred pain; these symptoms can interfere with work, daily activities, and patients’ quality of life (2, 3). Current neck-pain guidelines therefore emphasize education, active treatment, exercise, and self-management, whereas passive or pharmacological approaches alone are unlikely to achieve sustained recovery (3, 4).

The conceptual basis for chronic cervical pain has also broadened. Rather than attributing symptoms only to local structural injury, recent research increasingly considers dysfunction within the myofascial continuum (2, 5). Myofascial pain may occur as a distinct soft-tissue pain syndrome or coexist with other musculoskeletal conditions, and it can contribute to chronic pain through trigger-point activity, local hypersensitivity, and referred pain (2). Meanwhile, fascia is currently viewed less as an inert wrapping layer and more as a continuous, body-wide system involved in force transmission, sensory integration, and motor coordination (5–9). This perspective helps explain why symptoms experienced in the neck may be maintained by altered tension transmission or movement dysfunction involving the scapular girdle, thorax, or more distant soft-tissue networks (8, 9).

In traditional Chinese medicine (TCM), meridian sinew theory describes the linkage, constraint, and movement of muscles, tendons, bones, and joints and emphasizes knotting, aggregation, contracture, and urgency (10–14). Recent Chinese literature has used this theory to interpret neck-related disorders, emphasizing the identification of knotting sites and mechanical imbalance and the clinical principle of prioritizing the sinews in musculoskeletal dysfunction (10–14). The theory overlaps clinically with modern fascial-continuum concepts in its attention to pathway-based pain, tension-chain imbalance, and remote–local interactions, although the two systems should not be treated as anatomically identical (5–15).

Evidence for non-pharmacological care in chronic neck pain has also become more layered. Completed randomized trials suggest that tuina combined with Yijinjing may improve pain, function, and anxiety in non-specific chronic neck pain and that meridian sinew therapy may improve cervical extension dysfunction (15, 16). Systematic reviews and meta-analyses indicate that dry needling can offer short-term analgesic and functional benefits in mechanical neck pain, whereas Fascial Manipulation may reduce pain across several musculoskeletal conditions, with different levels of certainty across interventions (17, 18). Because most studies have examined these approaches separately, this review proposes a hypothesis-driven fascia–meridian sinew continuum framework to position them as distinct, potentially complementary functional nodes within a staged pathway (15–18).

## 2 Methods: narrative review and expert synthesis

This article was designed as a narrative review with expert synthesis rather than as a Preferred Reporting Items for Systematic Reviews and Meta-Analyses (PRISMA)-compliant systematic review, scoping review, or meta-analysis (19). Its purpose was to provide a transparent conceptual foundation for the proposed framework and to identify evidence-informed hypotheses for future research. We searched PubMed/MEDLINE, Web of Science Core Collection, Scopus, the Cochrane Library, Google Scholar, China National Knowledge Infrastructure (CNKI), and Wanfang from database inception to 20 June 2026. Both English- and Chinese-language sources were considered because the review draws on international fascial science, as well as Chinese-language literature, on meridian sinew theory. To reduce diagnostic ambiguity for primary care readers, the diagnostic terms used in this review were operationally defined before evidence synthesis. Chronic cervical myofascial pain (CCMP) was used as the primary diagnostic category of this manuscript, whereas myofascial pain syndrome, mechanical neck pain, and non-specific chronic neck pain were treated as overlapping but non-identical source labels for evidence synthesis (see Table 1).

**Table 1:** Operational diagnostic terminology used in this review.

Term	Operational scope in this review	Role in this manuscript
Chronic cervical myofascial pain (CCMP)	The primary diagnostic category: persistent or recurrent cervical pain dominated by myofascial features, including local tenderness or trigger-point-like nodal sites, taut-band or stiffness phenomena, restricted cervical or scapulothoracic movement, and possible referred pain, after exclusion of red flags or specific serious pathology.	Used as the main target condition and organizing construct for the fascia–meridian sinew framework.
Myofascial pain syndrome	A trigger-point-centered soft-tissue pain syndrome characterized by hyperirritable nodal sites in taut bands that may generate local and referred pain.	Used primarily when discussing trigger-point biology, microdialysis findings, and dry-needling mechanisms. It is treated as overlapping with, but not identical to, CCMP.
Mechanical neck pain	Neck pain provoked or modified by posture, movement, or loading, usually without serious structural disease. Trial populations labeled as mechanical neck pain may include CCMP-like phenotypes but are broader than CCMP.	Used when summarizing dry-needling and rehabilitation evidence from mechanical-neck-pain trials and meta-analyses.
Non-specific chronic neck pain	Chronic neck pain without a clearly identifiable specific structural pathology; it may include myofascial, postural, sensorimotor, and psychosocial contributors.	Used when summarizing trials of tuina plus Yijinjing and Jingjin therapy. It is broader than CCMP and is not assumed to be mechanistically homogeneous.

Certainty categories are qualitative and are not a formal GRADE assessment (20).

The search was organized around several blocks: chronic cervical myofascial pain and non-specific neck pain; fascia, the fascial continuum, myofascial continuity, fascial gliding, and hyaluronan; meridian sinew theory and Jingjin therapy; dry needling; Fascial Manipulation; Yijinjing and related mind–body exercise; primary-care implementation; adverse events; and objective mechanistic measures. Priority was given to systematic reviews, meta-analyses, clinical practice guidelines, completed randomized clinical trials, primary physiological or molecular studies, and high-quality methodological papers. Chinese-language studies were included when they addressed meridian sinew theory or clinical reasoning that was not adequately represented in English-language literature.

We included publications that addressed chronic neck pain, cervical myofascial pain, or related musculoskeletal pain mechanisms; reported evidence relevant to one of the four selected interventions; or clarified mechanistic, safety, implementation, or methodological issues central to the framework. We excluded inaccessible publications, non-scholarly commentaries, duplicates, studies unrelated to neck pain or myofascial mechanisms, and studies that made broad claims without clinical or mechanistic relevance. Study protocols were retained only to identify ongoing research and future directions and were not treated as completed clinical evidence. Because this was not a PRISMA-compliant systematic review, we did not calculate pooled effects or conduct formal risk-of-bias assessments. Instead, evidence certainty was described qualitatively, informed by the Grading of Recommendations Assessment, Development and Evaluation (GRADE) concepts but not presented as a formal GRADE assessment (20).

The four interventions were selected *a priori* because each represents a functional node in the proposed pathway. Dry needling corresponds to local trigger-point and hypersensitive-node desensitization; Fascial Manipulation corresponds to deep fascial gliding restoration and tension-chain reorganization; meridian sinew therapy corresponds to pathway-based identification and remote–local correction; and Yijinjing corresponds to active training, sensorimotor remodeling, and long-term maintenance (15–18, 21–23). This selection does not imply that other non-pharmacological interventions are unimportant or that it exhausts the treatment options for chronic cervical myofascial pain.

## 3 From meridian sinews to the fascial continuum: theoretical reframing

### 3.1 Core clinical implications of meridian sinew theory

If meridian sinew theory is presented only as a catalogue of classical descriptions, it remains difficult to connect with contemporary musculoskeletal medicine. For the purposes of this review, its clinical value is better captured as a soft-tissue framework organized around three ideas: pathway dependence, contracture-related dysfunction, and functional impairment (10–14). Pathway dependence refers to pain, tightness, and traction-related discomfort that follow anatomical routes or kinetic chains rather than remaining at a single painful point. Contracture-related dysfunction corresponds clinically to tension, spasm, tenderness, restricted sliding, and painful stretch. Functional impairment directs attention to movement limitation and disturbed dynamic balance, rather than pain alone (10–14).

This reframing allows meridian sinew theory to enter a more precise dialogue with modern musculoskeletal medicine. The dialogue should, however, remain cautious. Meridian sinew theory offers a clinically condensed language for distributed soft-tissue dysfunction; modern fascial science offers anatomical, biomechanical, and mechanobiological constructs that can be tested, refined, or challenged experimentally (5–9). Compatibility between the two languages is therefore useful; however, it should not be mistaken for anatomical identity.

### 3.2 Modern fascial continuum and myofascial continuity

Modern fascial research has moved beyond the view of fascia as a passive wrapping layer. It is increasingly understood as a continuous tissue system operating across anatomical scales (5–7). Evidence from fascial-system research, myofascial-chain reviews, and studies of intermuscular force transmission supports the possibility of mechanical coupling across muscles, aponeuroses, and fascial tissues (5, 8, 9). Primary *in vivo* data also support the plausibility of myofascial force transmission across tissue continuities, although its magnitude, direction, and clinical significance are likely to vary by region, loading condition, and patient phenotype (24).

Fascia is also a sensory and mechanobiological tissue. Hyaluronan in the deep fascia may facilitate interlayer sliding, whereas changes in hyaluronan concentration or aggregation may increase viscosity and restrict gliding (7, 25). Connective-tissue mechanotransduction studies show that mechanical stimuli can induce fibroblast cytoskeletal remodeling, offering a plausible cellular basis for tissue responses to needling, manual loading, or exercise (6, 21, 26). However, direct dose–response evidence in chronic cervical myofascial pain remains limited.

### 3.3 Epistemological status of the fascia–meridian sinew correspondence

The fascia–meridian sinew continuum proposed in this study is best understood as a clinical and theoretical correspondence, not as an anatomical equivalence or proven mechanistic homology. The correspondence operates at three levels. At the phenomenological level, both frameworks describe pathway-related pain, remote–local interaction, and functional restriction. At the clinical level, it may help organize palpation, movement assessment, and rehabilitation sequencing. At the research level, it generates testable questions about tissue gliding, tension transmission, sensorimotor control, and pain modulation. It does not establish a biological identity between meridian sinews and fascia.

**Figure 1:**
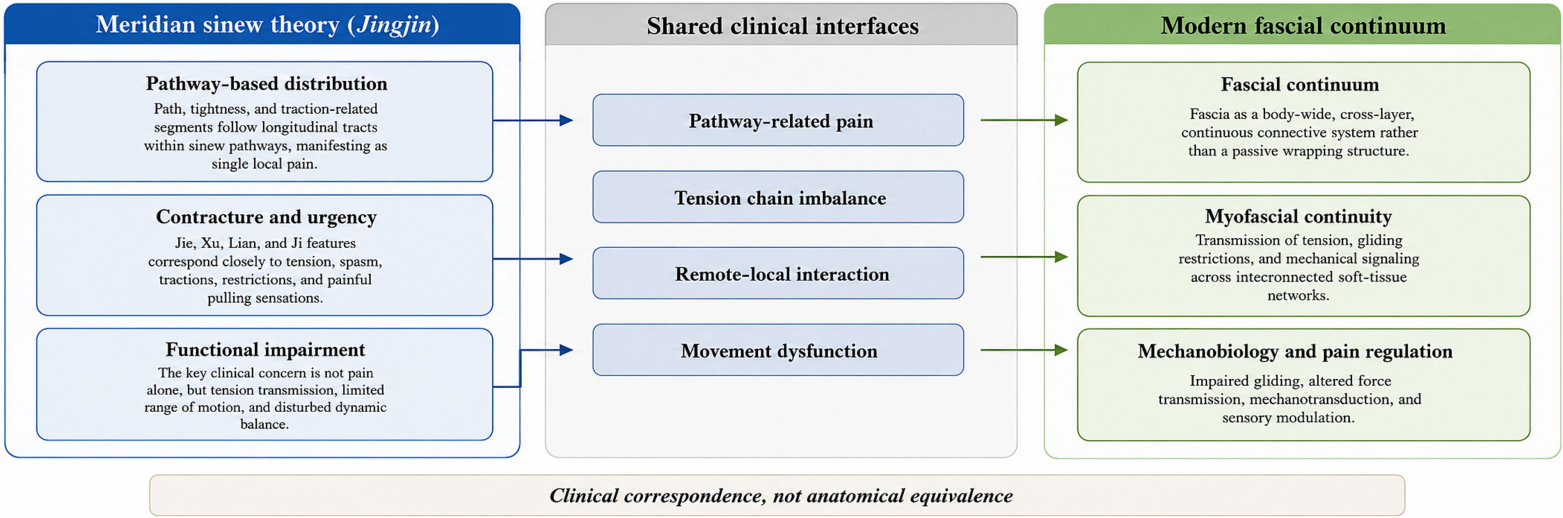
Conceptual framework linking meridian sinew theory and modern fascial science. The figure illustrates clinical correspondence rather than anatomical equivalence.

This distinction matters because it avoids circular reasoning. Fascial mechanobiology should not be used to “prove” meridian sinew theory, and meridian sinew theory should not be used to infer fascial mechanisms that have not been measured. A more productive approach is to let the two traditions generate hypotheses that can be examined with ultrasound elastography, surface electromyography, cervical kinematics, pressure pain thresholds, quantitative sensory testing, and patient-reported outcomes (27–29) (see Figure 1).

## 4 Shared pathophysiological basis of chronic cervical myofascial pain

Chronic cervical myofascial pain is unlikely to arise from a single local lesion. A more plausible account is a multilevel condition involving soft-tissue continuum dysfunction, local hypersensitive nodal sites, altered sensorimotor control, and dysregulated pain modulation (2–4, 30–41). For clarity, this review highlights four interacting domains: fascial densification and impaired gliding; trigger-point networks and local hyperalgesia; proprioceptive and motor-control disturbance; and peripheral–central coupling that contributes to chronicity.

### 4.1 Fascial densification and impaired gliding

Fascia-centered models suggest that myofascial pain may involve fibrosis, densification, and inflammatory activation of fascial tissue, although the directness of the evidence varies (4, 7, 25). Histological and biochemical studies support the role of hyaluronan in fascial-layer gliding, and altered hyaluronan behavior has been proposed to increase matrix viscosity and reduce interlayer sliding (7, 25). The causal pathway from hyaluronan aggregation to pain in chronic cervical myofascial pain is not fully established. At present, fascial densification is best regarded as a plausible mechanism that requires testing with imaging, tissue mechanics, and longitudinal clinical data (25, 27–29).

### 4.2 Trigger-point networks and local hyperalgesia

Myofascial trigger points provide a recognizable clinical expression of local hyperalgesia. Recent reviews describe myofascial pain syndrome as a chronic regional pain condition characterized by hyperirritable trigger points within taut bands, capable of producing both local and referred pain (2). Primary microdialysis studies have reported altered biochemical environments around active trigger points, including substance P and calcitonin gene-related peptide; animal studies of dry needling suggest that needling dosage can influence selected markers such as substance P, β-endorphin, and inflammatory mediators (32, 33). These findings support biological plausibility, but they do not yet establish a complete pathway from local tissue irritability to persistent clinical pain (32–34).

### 4.3 Disturbed proprioception, posture, and motor regulation

Chronic cervical myofascial pain also involves a mismatch between sensory input and motor output. A systematic review and meta-analysis found poorer joint position sense, oculomotor control, and postural stability in people with neck pain than in healthy controls (30). Reviews and randomized trials of sensorimotor training suggest that proprioception, balance, cervical range of motion, and disability may improve after targeted active training, supporting the view that sensorimotor disturbance is not merely secondary to pain but may help sustain symptoms and recurrence (36–38).

### 4.4 Peripheral–central coupling and chronicity

**Figure 2:**
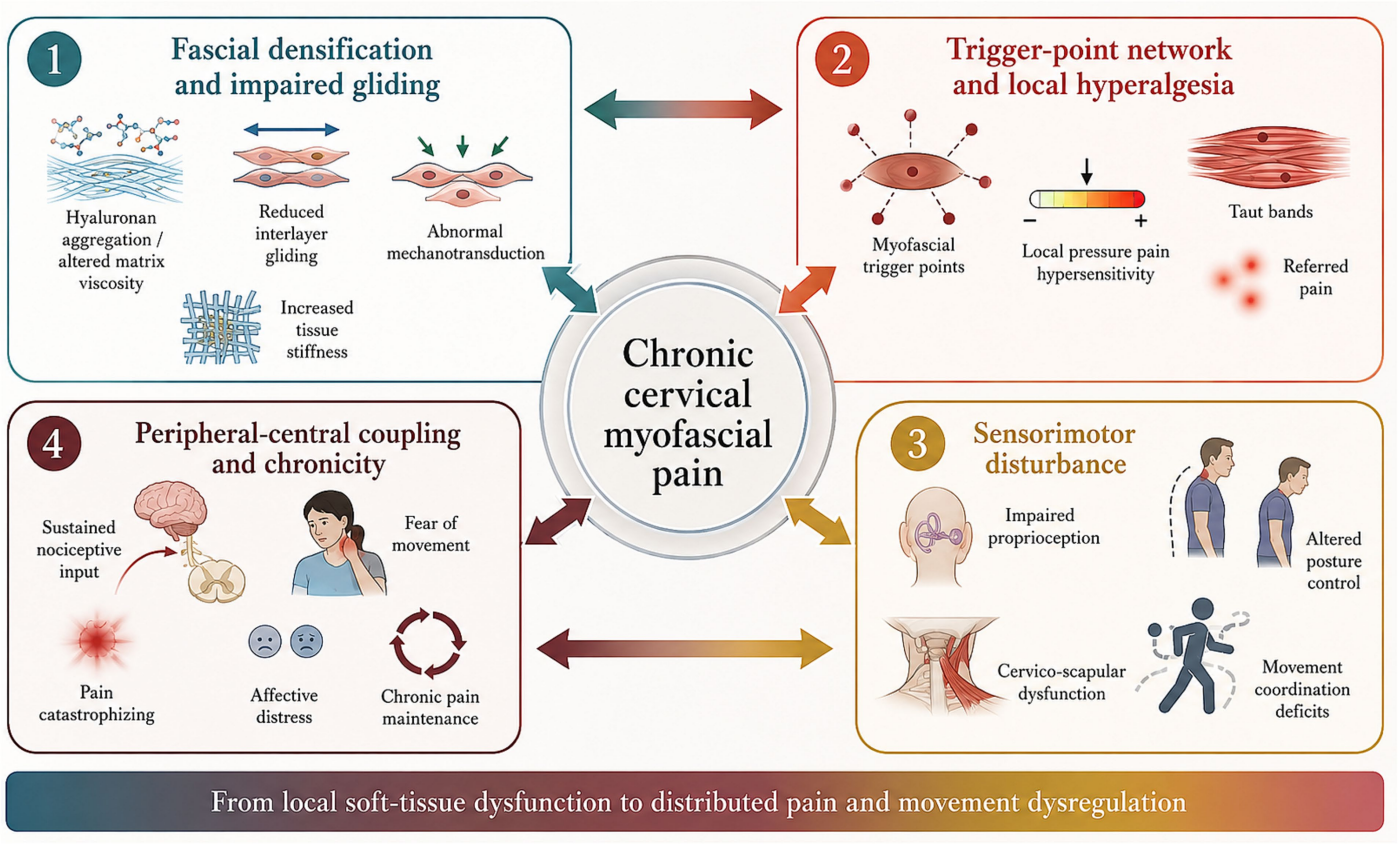
Multilevel pathophysiological network of chronic cervical myofascial pain. The four domains are proposed to interact bidirectionally and to reinforce symptom persistence.

Persistent soft-tissue abnormalities, trigger-point hypersensitivity, and sensorimotor disturbance may gradually interact with broader pain-modulation processes. Studies in chronic or recurrent neck pain have reported altered remote pain thresholds and associations between central sensitization inventory scores, kinesiophobia, catastrophizing, and anxiety (39, 40). A systematic review also identified pain catastrophizing and psychological distress as predictors of persistent or recurrent non-specific neck pain and related disability (41). Because the causal role of central sensitization in chronic pain remains debated, this review treats chronic cervical myofascial pain as a peripheral–central coupling process rather than a purely central disorder (26) (see Figure 2).

## 5 Layer-specific roles and evidence certainty for the four interventions

Within this pathophysiological framework, management of chronic cervical myofascial pain is better discussed as a set of potentially complementary strategies than as a contest over which technique is superior (17, 18, 42). The evidence base is uneven. Dry needling has relatively direct evidence for short-term outcomes in mechanical neck pain; meridian sinew therapy has emerging single-center randomized controlled trial (RCT) evidence; Fascial Manipulation is supported primarily by broader musculoskeletal pain data; and standalone Yijinjing remains under evaluation, although combined tuina plus Yijinjing has completed RCT evidence (15–18, 21–23).

The rationale for discussing these four interventions is functional. Dry needling represents local hypersensitive-node desensitization; Fascial Manipulation represents deep fascial gliding restoration and tension-chain reorganization; meridian sinew therapy represents pathway-based identification and management of remote–local dysfunction; and Yijinjing represents active training, sensorimotor remodeling, and long-term maintenance (15–18, 21–23). Table 2 summarizes the role assigned to each intervention in the proposed model and the main limitations of the supporting evidence.

**Table 2:** Evidence map and functional positioning of the four interventions.

Intervention	Functional node in the model	Main evidence base	Qualitative certainty and limitations	Implementation and safety notes
Dry needling	Local trigger-point/hypersensitive-node desensitization	Systematic reviews and meta-analyses in mechanical or myofascial neck/shoulder pain; primary mechanistic studies in trigger-point models	Moderate for short-term pain/PPT; low for long-term function and recurrence prevention	Skilled clinician required; risks include bleeding, bruising, infection, and rare pneumothorax in the cervicothoracic regions
Fascial Manipulation	Deep fascial gliding restoration and tension-chain reorganization	Systematic review/meta-analysis across musculoskeletal pain; scoping review; limited direct evidence from neck pain trials	Low; mostly indirect for chronic cervical myofascial pain	Requires specific training; avoid or refer in suspected malignancy, infection, acute inflammatory disease, fracture, or unstable vascular/neurological conditions
Meridian sinew therapy	Pathway-based recognition and remote–local chain correction	Single-center RCT in non-specific chronic neck pain with extension dysfunction; Chinese-language clinical literature	Low; promising but limited by setting, short follow-up, and operator dependence	Requires trained TCM/manual practitioners; caution with cervical instability, myelopathy, severe osteoporosis, or neurological red flags
Yijinjing	Active remodeling, sensorimotor retraining, and long-term maintenance	Completed RCT for tuina plus Yijinjing; standalone Yijinjing is currently being evaluated in protocols	Low–moderate for combined program; very low/pending for standalone Yijinjing	Generally low risk, but should be modified for severe pain flare, cervical instability, myelopathy, dizziness, or balance impairment

GRADE, Grading of Recommendations Assessment, Development and Evaluation; PPT, pressure pain threshold; RCT, randomized controlled trial; TCM, traditional Chinese medicine.

### 5.1 Dry needling

Dry needling maps most directly onto trigger-point networks and pressure hypersensitivity. Quantitatively, a 2025 meta-analysis of chronic mechanical neck pain found that dry needling improved pressure pain threshold [pooled mean difference (MD) 0.52, 95% confidence interval (CI) 0.39–0.65; *p* < 0.001] and Neck Disability Index score (pooled MD − 0.68, 95% CI − 1.32 to −0.05; *p* = 0.04), while effects on cervical range of motion were less consistent, supporting its role as short-term desensitization rather than complete rehabilitation (17). Evidence comparing deep and superficial dry needling points in the same direction: Short-term pain and disability benefits are plausible, but robust long-term data are lacking (21). Mechanistically, dry needling is likely to operate through local, spinal segmental, and supraspinal mechanisms rather than through a single pathway. At the local level, needle penetration or pistoning into a myofascial trigger point can elicit local twitch responses (LTRs), which are commonly interpreted as spinal reflex responses related to dysfunctional motor endplate activity; Hong’s clinical trial showed that immediate pain reduction was closely associated with elicitation of an LTR, regardless of whether lidocaine injection or dry needling was used (43). At the segmental level, dry-needle stimulation of a supraspinatus trigger point increased pressure pain thresholds at a neurologically related but untreated infraspinatus trigger point shortly after needling, supporting short-lived spinal antinociceptive modulation (44). At the supraspinal level, reduction of persistent peripheral nociceptive input may interact with descending inhibitory networks, including the periaqueductal gray–rostral ventromedial medulla pathways, which are particularly relevant in chronic pain states characterized by altered pain modulation and central sensitization (45). In parallel, animal studies suggest that dry needling can alter local biochemical mediators associated with pain and inflammation, and heat-conduction dry needling may influence transient receptor potential vanilloid 1 (TRPV1)/protein kinase C (PKC)/interleukin-6 (IL-6) signaling; however, TRPV1-mediated effects remain preliminary and should not be extrapolated as a proven human mechanism in chronic cervical myofascial pain (32, 33, 46).

### 5.2 Fascial Manipulation

Fascial Manipulation is conceptually associated with deep fascial gliding defects and tension-chain reorganization. A recent systematic review and meta-analysis found a significant pain-related effect favoring Fascial Manipulation over active-control interventions [effect size (ES) −0.80, 95% CI −1.30 to −0.29; *p* = 0.002; *I*^2^ = 85%], although the certainty of evidence was rated very low (18). A scoping review further describes the method’s focus on movement-unit assessment, painful patterns, and fascial points (22). Direct randomized evidence in chronic cervical myofascial pain is still scarce. Accordingly, claims that Fascial Manipulation restores hyaluronan viscosity or interlayer gliding should be treated as mechanistic hypotheses supported by fascial biology and preliminary clinical work, not as established clinical mechanisms (7, 25, 29).

### 5.3 Meridian sinew therapy

Meridian sinew therapy is a pathway-oriented clinical approach grounded in TCM. In a 2025 RCT of 160 patients with non-specific chronic neck pain and extension dysfunction, Jingjin therapy improved active cervical extension from 29.75° to 51.97° after six treatment sessions, but the between-group difference versus general manual therapy remained small and non-significant (MD −0.26°, 95% CI −2.62° to 2.09°; *p* = 0.828) (16). The study therefore supports feasibility and short-term functional improvement, but not superiority. This intervention is best presented as an emerging pathway-based approach that requires replication, protocol standardization, and longer follow-up.

### 5.4 Yijinjing

Yijinjing is best understood as an active mind–body exercise with potential relevance to sensorimotor remodeling and self-management. In a 2022 RCT, tuina combined with Yijinjing produced a greater week-8 reduction in visual analogue scale (VAS) pain score than tuina alone (between-group mean difference −1.2 points, 95% CI −1.6 to −0.8; *p* < 0.001), with a within-group VAS reduction of −5.4 points (95% CI −5.8 to −5.1) in the combined-treatment group and benefits maintained at 12 weeks (15). However, the trial tested a combined program rather than a standalone Yijinjing. Ongoing trials of Yijinjing alone should therefore be read as future research, not as completed evidence (23).

## 6 Hypothesis-driven staged model: from release to remodeling

The staged model proposed here remains a hypothesis-driven theoretical framework awaiting clinical validation. It draws on mechanistic inference, indirect evidence, and expert synthesis; no clinical trial has directly tested the entire sequence. It should therefore be used as a research framework and a clinical reasoning aid, not as a prescriptive treatment algorithm.

In the first stage, release and desensitization may be most relevant when trigger-point hyperexcitability, pressure hypersensitivity, and protective guarding are prominent. On the basis of indirect evidence, dry needling or local meridian sinew/fascial release may reduce pain-related inhibition and make movement more tolerable (17, 21). In the second stage, chain correction and gliding restoration may become more relevant when symptoms persist despite local improvement or when remote–local coupling, scapulothoracic involvement, and wider tension-chain imbalance are evident. In this study, Fascial Manipulation and meridian sinew therapy may help trained clinicians identify restricted sites and abnormal transmission patterns along continuous networks (16, 18, 22).

**Figure 3:**
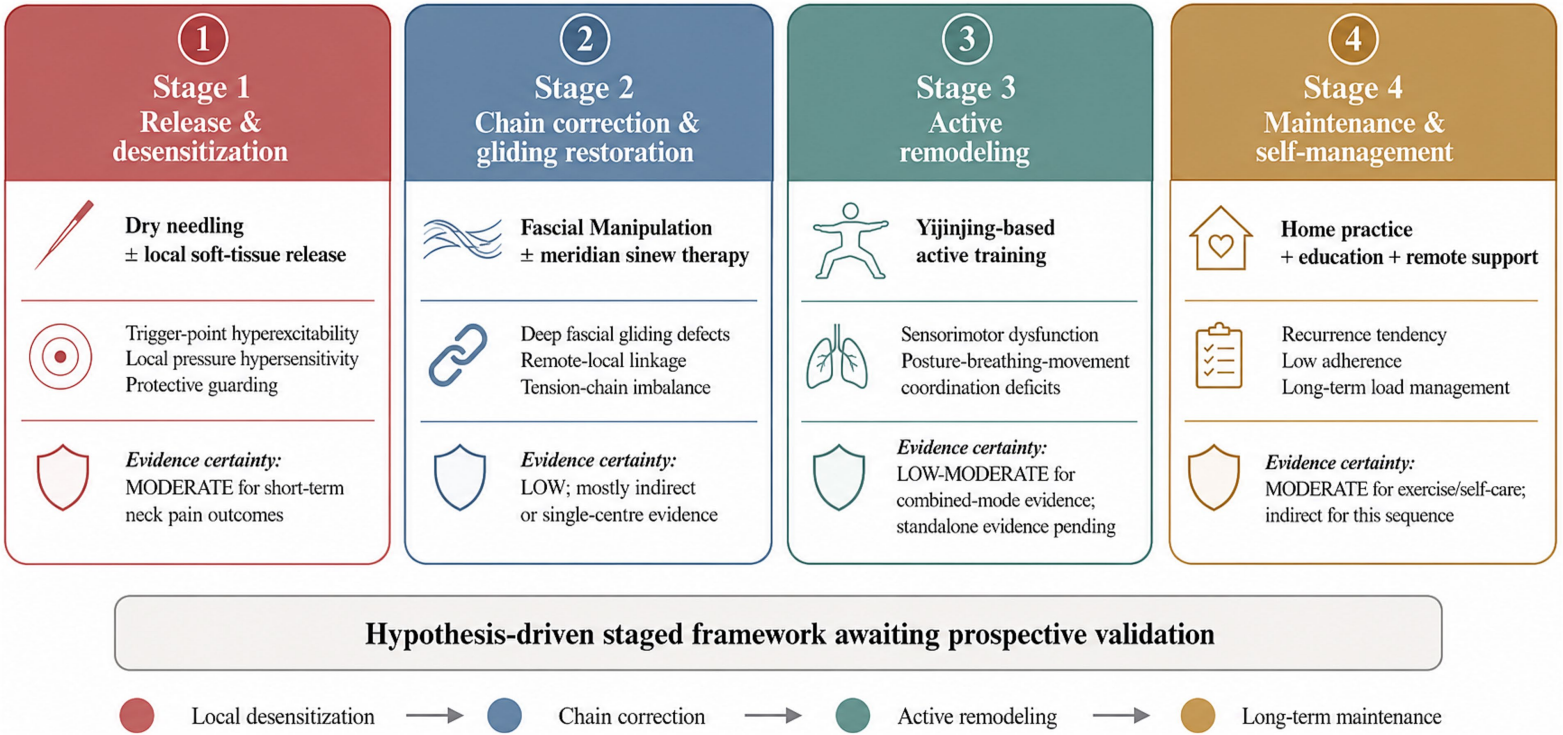
Hypothesis-driven staged framework linking four representative interventions to functional nodes. Evidence certainty is shown qualitatively for each stage; the sequence itself has not yet been validated in a prospective trial.

The third stage, active remodeling, may be considered once pain has decreased enough for the patient to participate in active loading. Yijinjing is one possible approach because it combines posture, breathing, and controlled movement, although the strongest evidence at present concerns combined tuina plus Yijinjing rather than Yijinjing alone (15, 23). The fourth stage, maintenance and self-management, is supported by broader neck-pain evidence showing that exercise, self-care education, telerehabilitation, and mobile health interventions can improve outcomes or reduce recurrence risk. This evidence supports the components of long-term management, but it does not validate the specific four-stage sequence proposed here (42, 47–50) (see Figure 3).

## 7 Clinical reasoning, transferability, and safety

### 7.1 Phenotype-informed clinical reasoning guide

The stratification proposed in this review is a clinical reasoning guide based on expert inference, not a validated decision algorithm. In real-world care, treatment decisions are shaped by patient preference, cost, insurance coverage, practitioner availability, health literacy, cultural acceptability, and comorbidities. These considerations are particularly important in international and non-TCM settings, where meridian sinew therapy or Yijinjing may be unfamiliar or unavailable (see Table 3).

**Table 3:** Phenotype-informed clinical reasoning guide.

Phenotype (working construct)	Clinical features	Possible reasoning pathway	Evidence caveat
Local trigger-point dominance	Marked local tenderness, pressure hypersensitivity, and pain-limited movement	Consider local desensitization first; refer for dry needling only when trained providers and appropriate safety screening are available	Indirect reasoning supported by dry-needling evidence; not a validated rule
Continuum or chain-dominant presentation	Persistent symptoms, scapular/thoracic involvement, remote–local linkage, limited response to local care	Consider assessment of scapulothoracic mechanics, movement chains, and referral to trained Fascial Manipulation/traditional Chinese medicine manual clinicians when appropriate	Indirect reasoning; direct comparative trials are absent
Sensorimotor or recurrence-dominant presentation	Frequent recurrence, low activity level, sedentary behavior, poor postural control, fear of movement	Prioritize active exercise, self-management, and culturally adaptable Yijinjing or equivalent movement training	Supported by broader exercise/self-care evidence, Yijinjing-specific data is limited

This table is intended for hypothesis generation and clinical discussion, not as a validated decision algorithm.

### 7.2 Transferability to primary care and non-TCM systems

In primary care, the framework is most useful as a triage and referral logic rather than as a requirement that all clinicians deliver all four interventions. General practitioners and primary-care clinicians can screen for red flags, identify dominant clinical patterns, provide education, encourage graded activity, monitor response, and refer to appropriately trained practitioners when specialized manual or needling interventions are indicated (3, 31).

Yijinjing may be transferable beyond Chinese cultural settings if it is operationalized as a structured, low- to moderate-intensity mind–body exercise focused on posture, breathing, and controlled movement. International implementation would require standardized translated instructions, culturally neutral explanations, remote supervision or video-based teaching, and modifications for patients with limited mobility or low exercise confidence (15, 42, 47–50). Meridian sinew therapy is less readily transferable because it depends on TCM-specific assessment and manual skills. In non-TCM systems, its concepts may still inform a general clinical heuristic: assessing remote–local linkages, pathway-related tenderness, and movement-chain dysfunction, with referral to trained practitioners when available. Fascial Manipulation also requires dedicated training; when such expertise is absent, primary-care clinicians should avoid specialized procedures and focus instead on referral, monitoring, and active self-management.

### 7.3 Safety, contraindications, and referral considerations

Safety deserves explicit attention in any clinical review aimed at primary care. Dry needling is usually associated with minor adverse events such as bleeding, bruising, and post-needling pain, but rare serious events, including pneumothorax, infection, hematoma, and nerve injury, have been reported, particularly in the cervicothoracic regions (51–53). It should therefore be performed only by credentialed practitioners who use sterile technique, obtain informed consent, and complete appropriate anatomical and risk screening.

Fascial Manipulation and meridian sinew-based manual therapies may be inappropriate for or may require medical referral in patients with suspected fracture, malignancy, infection, acute inflammatory disease, severe osteoporosis, vascular compromise, progressive neurological deficit, or cervical myelopathy (54, 55). Yijinjing is generally low risk, but it should be modified or deferred during severe pain flares and in patients with marked dizziness, cervical instability, myelopathy, acute trauma, or significant balance impairment. Across all four approaches, worsening neurological symptoms, gait disturbance, bowel or bladder dysfunction, unexplained weight loss, fever, a history of cancer, or signs of cervical myelopathy should prompt medical evaluation rather than continued manual or exercise-based treatment (3, 55).

### 7.4 Population and intervention heterogeneity

Chronic cervical myofascial pain is heterogeneous. Patients differ in chronicity, pain sensitivity, age, sex, psychosocial context, comorbidity, and cultural background. Women are overrepresented in many neck-pain cohorts, and older adults may have coexisting degenerative cervical disease, osteoporosis, or neurological compromise that may alter the balance of risks and benefits (1, 15, 30, 31). Patients with high catastrophizing, anxiety, kinesiophobia, or widespread sensitivity may need a stronger emphasis on education, graded exposure, and psychologically informed rehabilitation (39–41, 47). Future studies should assess whether the proposed framework performs differently according to sex, age, chronicity, sensitization profile, comorbidity, and care setting.

## 8 Evidence gaps and future research agenda

Several gaps must be addressed before the proposed staged framework can be translated into routine practice. First, disease definitions and outcome domains remain inconsistent. Terms such as cervical myofascial pain, mechanical neck pain, non-specific chronic neck pain, and myofascial pain syndrome are often used interchangeably, and trials vary widely in outcome selection (31, 56, 57). Greater harmonization of diagnostic definitions, core outcome domains, and patient-reported outcome measures (PROMs) is needed.

Second, intervention reporting and fidelity remain weak. Studies of exercise, manual therapy, and dry needling often omit important details on practitioner expertise, materials, dose, adherence, and fidelity, which limits replication (58–61). Future trials should follow the Template for Intervention Description and Replication (TIDieR) and Consensus on Exercise Reporting Template (CERT) reporting frameworks and prospectively monitor adherence and treatment fidelity (60, 61). Third, mechanistic specificity is still limited. Future studies should include objective measures such as ultrasound elastography, fascial thickness and gliding assessment, pressure pain thresholds, quantitative sensory testing, surface electromyography, proprioceptive tests, inertial measurement units for cervical kinematics, and wearable sensor monitoring (27–29, 62–64).

Fourth, protocols should not be interpreted as evidence of effectiveness. Completed combined-mode trials provide preliminary clinical context, whereas ongoing protocols evaluating standalone Yijinjing should be cited only as future research (15, 23). Fifth, the field needs head-to-head and sequencing trials. A decisive test would compare the proposed staged protocol with each intervention in isolation and with non-sequenced multimodal care. Such trials should include formal evidence grading, mechanistic substudies, long-term recurrence outcomes, adverse-event monitoring, health-economic analysis, and patient-reported experience measures alongside PROMs (65), and implementation outcomes relevant to primary care.

## 9 Limitations of this review

Several limitations should be acknowledged. First, this article is a narrative review with expert synthesis rather than a systematic review or meta-analysis. Although the search process is documented, we did not conduct exhaustive screening, formal risk-of-bias assessment, or quantitative synthesis. Second, the four interventions were selected for their theoretical and clinical representativeness within the proposed pathway; they do not exhaust all non-pharmacological treatment options for chronic cervical myofascial pain. Third, the evidence base is uneven: dry needling and combined tuina–Yijinjing have more direct clinical evidence than meridian sinew therapy, Fascial Manipulation specifically for neck pain, or standalone Yijinjing. Fourth, several mechanistic links discussed here, particularly the correspondence between meridian sinew theory and the fascial continuum and the proposed layer-specific effects of interventions, remain hypotheses based on indirect evidence. Finally, the staged synergistic model has not been prospectively validated, and its applicability across health systems, cultures, and patient phenotypes remains uncertain.

## 10 Conclusion

Meridian sinew theory and modern fascial science should not be treated as one-to-one equivalents. They do, however, converge in clinically relevant domains, including pathway-based pain, tension-chain imbalance, remote–local interaction, and functional restriction. Chronic cervical myofascial pain can therefore be conceptualized as a multilevel disorder involving local hypersensitive nodes, impaired fascial gliding, tension-chain imbalance, sensorimotor dysfunction, and pain-modulation processes. Within this framework, dry needling, Fascial Manipulation, meridian sinew therapy, and Yijinjing may be viewed as representative interventions acting at different functional nodes. The staged model proposed in this study, from release to remodeling, is a hypothesis-driven theoretical framework rather than a validated clinical algorithm. Its value lies in generating testable questions for integrative rehabilitation, primary-care implementation, and future mechanistic trials. Harmonized definitions, standardized interventions, objective mechanistic measures, safety reporting, and prospective staged trials are needed before the framework can be translated into routine care.
