# Supplementary material for: A review of the synergistic mechanisms of Yijinjing, meridian sinew therapy, dry needling, and Fascial Manipulation in chronic cervical myofascial pain based on the fascia–meridian sinew continuum
Source: Front Med (Lausanne). 2026 Jul 23;13:1881739. doi: 10.3389/fmed.2026.1881739 (PMC13441923; doi:10.3389/fmed.2026.1881739)
Supplement: Supplementary file 1 [file Data_Sheet_1.docx]

# Search strategy and article-selection rationale

This document provides the search strategy used to revise the narrative review with expert synthesis entitled “A review of the synergistic mechanisms of Yijinjing, meridian sinew therapy, dry needling and Fascial Manipulation for chronic cervical myofascial pain based on the fascia–meridian sinew continuum”. The review was not designed as a systematic review or scoping review. The strategy below is reported to improve transparency and reproducibility, to clarify the basis for article selection, and to distinguish completed clinical evidence, protocols, mechanistic evidence and expert synthesis.

## 1 Databases and search date

| Database/source | Coverage and purpose | Search date |
| --- | --- | --- |
| PubMed/MEDLINE | Biomedical literature, MeSH-based searches, RCTs, systematic reviews, mechanistic studies, adverse events, clinical practice guidelines | Updated 20 June 2026 |
| Web of Science Core Collection | Citation tracking and multidisciplinary rehabilitation/fascia literature | Updated 20 June 2026 |
| Scopus | Citation tracking and rehabilitation/manual therapy literature | Updated 20 June 2026 |
| Cochrane Library | Systematic reviews and controlled trial records | Updated 20 June 2026 |
| Google Scholar | Forward/backward citation checking and grey bibliographic verification | Updated 20 June 2026 |
| CNKI (China National Knowledge Infrastructure) | Chinese-language literature on 经筋学说, 经筋疗法, 易筋经 and related TCM clinical reasoning | Updated 20 June 2026 |
| Wanfang Data | Supplementary Chinese-language search for TCM and rehabilitation literature | Updated 20 June 2026 |

## 2 PubMed MeSH terms and free-text terms

| Concept block | MeSH terms | Free-text terms |
| --- | --- | --- |
| Condition | "Neck Pain"[Mesh]; "Myofascial Pain Syndromes"[Mesh]; "Musculoskeletal Pain"[Mesh]; "Chronic Pain"[Mesh] | chronic cervical myofascial pain; cervical myofascial pain; chronic neck pain; non-specific chronic neck pain; nonspecific chronic neck pain; mechanical neck pain; cervicalgia; neck disability; trigger point neck pain |
| Fascia and continuum mechanisms | "Fascia"[Mesh]; "Connective Tissue"[Mesh]; "Hyaluronic Acid"[Mesh]; "Mechanotransduction, Cellular"[Mesh]; "Proprioception"[Mesh] | fascial continuum; fascial system; myofascial continuum; myofascial continuity; myofascial chain; fascial gliding; fascial densification; hyaluronan; hyaluronic acid; epimuscular force transmission; mechanobiology; mechanotransduction; sensorimotor control |
| Dry needling | "Acupuncture Therapy"[Mesh]; "Needles"[Mesh]; "Trigger Points"[Mesh] | dry needling; trigger point dry needling; myofascial trigger point needling; deep dry needling; superficial dry needling; local twitch response; pressure pain threshold; calcitonin gene-related peptide; substance P; beta-endorphin |
| Fascial Manipulation | "Musculoskeletal Manipulations"[Mesh]; "Physical Therapy Modalities"[Mesh] | Fascial Manipulation; Stecco method; fascial therapy; myofascial release; fascia manual therapy; deep fascia; deep fascial gliding defects; chain correction |
| Meridian sinew theory and therapy | "Medicine, Chinese Traditional"[Mesh]; "Meridians"[Mesh]; "Manipulation, Chinese Traditional"[Mesh] | meridian sinew; Jingjin; Jingjin therapy; meridian sinew therapy; sinew meridian; meridian sinew tuina; pathway-based manual therapy; remote-local coupling |
| Yijinjing and active training | "Exercise Therapy"[Mesh]; "Qigong"[Mesh]; "Tai Ji"[Mesh]; "Mind-Body Therapies"[Mesh]; "Self-Management"[Mesh] | Yijinjing; Yi Jin Jing; Yijin Jing; Qigong exercise; mind-body exercise; cervical functional training; sensorimotor training; active remodeling; home exercise; adherence |
| Implementation and safety | "Primary Health Care"[Mesh]; "Family Practice"[Mesh]; "Referral and Consultation"[Mesh]; "Patient Safety"[Mesh]; "Adverse Events"; "Contraindications" | primary care; family medicine; implementation; transferability; cultural adaptation; training; adverse events; contraindications; pneumothorax; infection; hematoma; cervical myelopathy; cervical instability |
| Digital monitoring and future research | "Wearable Electronic Devices"[Mesh]; "Mobile Applications"[Mesh]; "Telemedicine"[Mesh]; "Electromyography"[Mesh]; "Ultrasonography"[Mesh] | wearable sensor; inertial measurement unit; IMU; surface EMG; cervical kinematics; ultrasound elastography; shear-wave elastography; mHealth; telerehabilitation; patient-reported experience measures; PREMs; health economics |

## 3 Example PubMed search strings

The following strings were adapted according to database syntax. Date filters were not applied initially; more recent evidence was prioritized during interpretation when several sources addressed the same question.

| Search purpose | Example search string |
| --- | --- |
| Core condition and fascia mechanisms | ("Neck Pain"[Mesh] OR "Myofascial Pain Syndromes"[Mesh] OR chronic neck pain[tiab] OR cervical myofascial pain[tiab] OR mechanical neck pain[tiab]) AND ("Fascia"[Mesh] OR fascial continuum[tiab] OR myofascial continuity[tiab] OR myofascial chain[tiab] OR fascial gliding[tiab] OR hyaluronan[tiab] OR mechanotransduction[tiab]) |
| Dry needling evidence and mechanisms | ("Neck Pain"[Mesh] OR chronic neck pain[tiab] OR mechanical neck pain[tiab]) AND (dry needling[tiab] OR trigger point needling[tiab]) AND (systematic review[pt] OR meta-analysis[pt] OR randomized controlled trial[pt] OR pressure pain threshold[tiab] OR substance P[tiab] OR calcitonin gene-related peptide[tiab]) |
| Fascial Manipulation evidence | (Fascial Manipulation[tiab] OR Stecco[tiab] OR deep fascial gliding[tiab] OR fascia manual therapy[tiab]) AND (pain[tiab] OR musculoskeletal pain[tiab] OR neck pain[tiab]) |
| Meridian sinew and Jingjin therapy | ("Medicine, Chinese Traditional"[Mesh] OR "Meridians"[Mesh] OR meridian sinew[tiab] OR Jingjin[tiab] OR meridian sinew therapy[tiab]) AND (neck pain[tiab] OR cervical pain[tiab] OR chronic neck pain[tiab]) |
| Yijinjing and active training | (Yijinjing[tiab] OR Yi Jin Jing[tiab] OR Qigong[Mesh] OR qigong[tiab] OR mind-body exercise[tiab]) AND (neck pain[tiab] OR chronic neck pain[tiab] OR cervical pain[tiab]) |
| Safety and implementation | (dry needling[tiab] OR Fascial Manipulation[tiab] OR manual therapy[tiab] OR Yijinjing[tiab] OR Jingjin[tiab]) AND (adverse events[tiab] OR safety[tiab] OR contraindications[tiab] OR pneumothorax[tiab] OR primary care[tiab] OR implementation[tiab]) |
| Objective monitoring | ("Neck Pain"[Mesh] OR chronic neck pain[tiab]) AND (wearable[tiab] OR inertial measurement unit[tiab] OR IMU[tiab] OR surface electromyography[tiab] OR ultrasound elastography[tiab] OR shear-wave elastography[tiab] OR sensor[tiab]) |

## 4 CNKI and Wanfang Chinese search terms

| Concept block | Chinese terms used |
| --- | --- |
| Condition | 颈痛；慢性颈痛；颈项痛；颈项肌筋膜疼痛；肌筋膜疼痛综合征；非特异性慢性颈痛；机械性颈痛；颈椎病；颈源性头痛 |
| Meridian sinew theory | 经筋；经筋学说；十二经筋；经筋失衡；结筋病灶点；筋骨失衡；以筋为先；筋膜链；经筋与筋膜；经筋辨证 |
| Meridian sinew therapy | 经筋疗法；经筋推拿；经筋治疗；经筋路径；循经触诊；远端取穴；远近联动；链式纠偏 |
| Yijinjing | 易筋经；健身气功易筋经；易筋经功法；易筋经训练；易筋经联合推拿；身心运动；主动训练；功能重塑 |
| Dry needling | 干针；干针疗法；肌筋膜触发点；触发点针刺；压痛阈；局部抽搐反应 |
| Fascial Manipulation and fascia | Fascial Manipulation；筋膜手法；筋膜松解；深筋膜；筋膜滑移；筋膜致密化；透明质酸；肌筋膜连续性；筋膜连续体 |
| Implementation and safety | 基层医疗；全科医学；家庭医学；非药物干预；不良事件；禁忌证；安全性；依从性；远程康复；可推广性 |

## 5 Inclusion and exclusion criteria

| Included | Excluded |
| --- | --- |
| Systematic reviews, meta-analyses and completed randomized controlled trials relevant to chronic neck pain, cervical myofascial pain, dry needling, Fascial Manipulation, meridian sinew therapy, Yijinjing, sensorimotor training or self-management. | Non-scholarly webpages, duplicated records, abstracts without sufficient methodological information, and papers unrelated to neck pain, myofascial pain, fascia, meridian sinew theory or the four interventions. |
| Primary molecular, histological, imaging, biomechanical or neurophysiological studies relevant to fascial gliding, hyaluronan, mechanotransduction, trigger-point biochemistry, proprioception or sensor-based outcomes. | Studies making broad theoretical claims without clinical or mechanistic relevance to the proposed framework. |
| Clinical practice guidelines, implementation papers, safety/adverse event studies and reporting-methodology papers relevant to primary care translation and intervention standardization. | Protocol papers as direct evidence of effectiveness; these were retained only to identify ongoing trials and future research directions. |
| Chinese-language literature from CNKI/Wanfang when it addressed meridian sinew theory, Jingjin therapy, Yijinjing or TCM-specific clinical reasoning. | Chinese-language papers not indexed in CNKI/Wanfang or lacking sufficient bibliographic information. |

## 6 Basis for article selection and evidence interpretation

Article selection was guided by relevance to the conceptual framework and by evidentiary role. Completed RCTs and meta-analyses were treated as clinical evidence; primary molecular, physiological, imaging or biomechanical studies were used to support mechanistic plausibility; scoping reviews and narrative reviews were used to map concepts and identify gaps; protocols were cited only as evidence of ongoing research, not as evidence of efficacy. When multiple papers addressed the same claim, more recent systematic reviews, completed RCTs, clinical practice guidelines and primary mechanistic studies were prioritized over opinion pieces or lower-level narrative sources.

Evidence certainty in the manuscript was described qualitatively rather than by a formal GRADE process because this review was not designed as a systematic review. The categories used were: moderate (several completed studies or systematic reviews with reasonably direct evidence but residual limitations); low (limited, indirect, heterogeneous or single-centre evidence); and very low/pending (protocol-only, hypothesis-driven or primarily mechanistic inference).

## 7 How reviewer concerns were addressed through the search strategy

| Reviewer concern | Search/selection response |
| --- | --- |
| Methodological opacity | A dedicated Methods section and this supplementary search-strategy document were added. |
| Protocol papers cited as evidence | Protocols were reclassified as ongoing/future research and were not used as evidence of clinical effectiveness. |
| Lack of primary mechanistic evidence | Searches explicitly targeted molecular/physiological evidence on trigger-point biochemistry, hyaluronan, mechanotransduction, force transmission, ultrasound and sensor-based outcomes. |
| Primary-care transferability | Searches included primary care, implementation, telerehabilitation, mHealth, safety and contraindications. |
| Safety omissions | Searches included adverse events, contraindications, pneumothorax, infection, hematoma, cervical myelopathy and manual therapy safety. |
| Evidence certainty | A qualitative evidence map was added to the manuscript and figure evidence indicators were revised. |
